# Supplementary material for: Implementation of an open-source robotic platform for SARS-CoV-2 testing by real-time RT-PCR
Source: PLoS One. 2021 Jul 14;16(7):e0252509. doi: 10.1371/journal.pone.0252509 (PMC8279358; doi:10.1371/journal.pone.0252509)
Supplement: S1 File — Expanded methodology for the height calculation function and description of the platforms used in the EMQN comparison. (DOCX) [file pone.0252509.s001.docx]

***Calculation of aspiration height of the OT-2 pipettes***

A common technique to manually pipette is to plunge the tip a few mm below the meniscus. We risk aspirating air if we plunge too little and too much immersion can cause some liquid to cling to the outside of the tip. However, OT-2 stations do not have conductive tips to detect a first contact with liquid. To amend this problem, we developed a function that calculates the most suitable aspiration height for the pipette based on the remaining volume available and the shape of the container (see Methods). It also considers that a reagent can be in multiple sources and switches to another source if there is not enough volume in the current one (i.e. different screwcaps). This is useful to increase volume accuracy, to avoid overspills, and to use limiting reagents efficiently. In Figure S1, we show a simulation of a common scenario where we have two screwcaps with a limiting reagent. Initial volumes can be adjusted using simulations to minimize reagent leftover.

**Figure S1. Simulations using the height calculation function.** The plot represents two 2 ml screwcaps with a reagent that is used in 96 wells (one full plate), where 20 µl are dispensed in each one. The initial reagent is2112 µl and includes an initial 10% extra volume (20 µl * 96 samples * 1.1) to compensate for pipette inaccuracies. The left y axis (green) represents the remaining volume in each iteration after aspirating 20 µl. The right x axis (dark grey) indicates the height from where we are aspirating. The red dotted line depicts the volume that fits in the screwcap cone (50 µl). If there is less volume than the needed aspirated volume (20 µl) the system will switch to the next reagent location (screwcap 2).

**rRT-PCR using Roche Modular Kit**

An automatic nucleic acid extraction from 400 µl of inactivated samples was performed on a MagNa Pure Compact instrument (Roche Applied Science, Mannheim, Germany) using the MagNA Pure Compact Nucleic Acid Isolation Kit I - Large Volume (Roche), following the manufacturer’s instructions. Equine arteritis virus (EAV), a positive-sense single-stranded RNA virus, was added to all samples prior to RNA extraction and served as an internal extraction and amplification control. For detection of SARS-CoV-2, a 76 bp long fragment from the E gene was amplified with specific primers and detected with a FAM label hydrolysis probe using a LightMix Modular kit (Roche). The assay detects SARS and 2019-nCoV pneumonia virus (bat-associated SARS related Sarbecovirus). RT-PCR was performed in combination with the LightCycler® Multiplex RNA Virus Master (Roche) on the LightCycler 480 Real-Time PCR System (Roche) following recommended cycling conditions: reverse transcription at 55 °C for 3 min, and 95 °C for 20 sec, followed by 45 cycles of PCR at 95 °C for 3 sec and 60 °C for 30 sec (Corman).

**rRT-PCR using COBAS 6800**

Cobas 6800 (Roche) is a fully automated sample-to-result platform, including the sample supply module, the transfer module, the processing module, and the analytic module. For detection of SARS-CoV-2, a two-target RT-PCR is used: one targeting ORF1, a non-structural region that is unique to SARS-CoV-2 (target 1), and the second targeting a conserved region in the structural protein envelope E gene for pan-Sarbecovirus detection (target 2). The pan-Sarbecovirus primers and probe should also detect the SARS-CoV-2 virus. The test utilizes RNA internal control for sample preparation and PCR amplification process control. Automated data management was performed by the manufacturer’s software, which assigns test results for all tests. In our study, 400 µl of aliquots with lysis buffer were extracted on the Cobas 6800 system and tested following the manufacturer’s instructions. Testing was performed in batches of 94 samples plus one negative and positive control each.

**rRT-PCR using SeeGene**

RNA was extracted using Seegene STARMag 96 X 4 Universal Cartridge Kit (Seegene) on the Microlab STAR Liquid Handling System (Hamilton). rRT-PCR protocol with the Allplex™ 2019-nCoV Assay was automated: prepared on the Microlab STAR Liquid Handling System (Hamilton) and detected on the CFX96 Touch Deep Well Real-Time PCR Detection System (Bio-Rad). C_t_ from FAM (E gene), Cal Red 610 (RDRP gene), Quasar 670 (N gene) and HEX (internal control) were acquired.

**Interpretation of results**

A sample was considered negative if the internal control was amplified but not the viral genes. A specimen was considered invalid when there was no amplification of the internal control. Samples were considered positive when a signal was detected at C_t_<38 for Roche (Modular and COBAS 6800) or Ct<37 for any gene in case of Seegene technique.
