# Supplementary material for: Implementation of an open-source robotic platform for SARS-CoV-2 testing by real-time RT-PCR
Source: PLoS One. 2021 Jul 14;16(7):e0252509. doi: 10.1371/journal.pone.0252509 (PMC8279358; doi:10.1371/journal.pone.0252509)
Supplement: S2 File — Description of OT-2 stations including the labware placement for every circuit described in this manuscript. (PDF) [file pone.0252509.s002.pdf]

OT2-KF station configurations

Summary

This supplementary material contains the pipette configuration, materials and disposition for the stations included in the OT2-KF Pathogen circuit and the OT2-KF Viral Pathogen II (VPII) circuit.
OT2-KF Pathogen and Viral Pathogen II share stations A and C labwares and only differ in stations B1 and B2.
The total material needed for either circuit is at the end of the supplementary material.

OT2-KF Station A

Sample
preparation

KF-A

Disposition

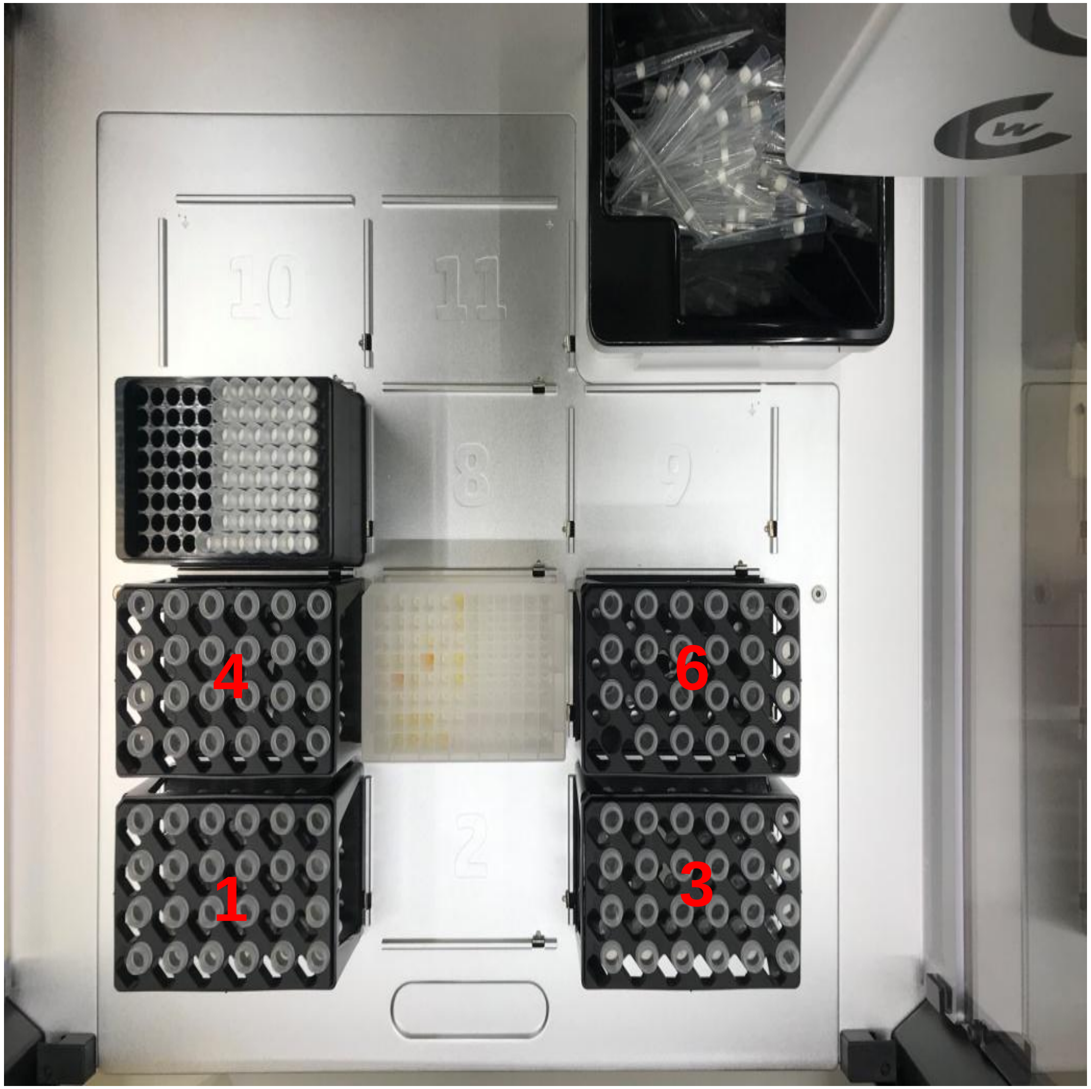

Pipettes

Left: p1000

Right: p20

Slots

1. Samples-1 (2ml)
2. Tiprack 20
3. Samples-2 (2ml)
4. Samples-3 (2ml)
5. KingFisher Deepwell plate
6. Samples-4 (2ml)
7. Tiprack P1000
8. *Empty*
9. *Empty*
10. *Empty*
11. *Empty*
12. Wastebin

OT2-KF Pathogen Station B1

Reagent setup
(plate filling)

KF-B1 Disposition pathogen

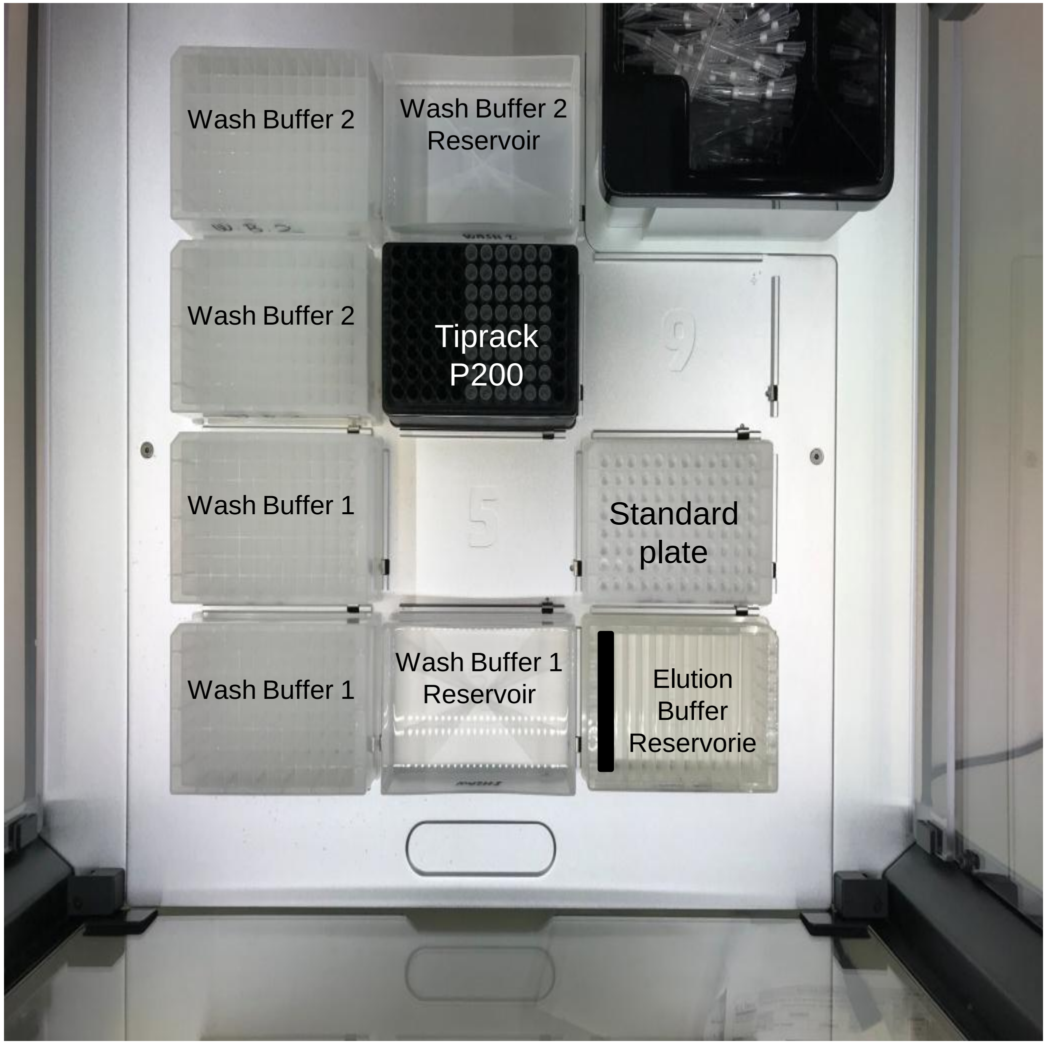

Pipettes
Left: m20 (not used)
Right: m300

Slots

1. KingFisher DeepWell plate
2. 300ml NEST reservoir
3. 12 channel NEST reservoir
4. KingFisher DeepWell plate
5. *Empty*
6. KingFisher Standard plate
7. KingFisher DeepWell plate
8. Tiprack 200
9. *Empty*
10. KingFisher DeepWell plate
11. 300ml NEST reservoir
12. Wastebin

OT2-KF Pathogen Station B2

Sample preparation

KF-B2

Disposition pathogen

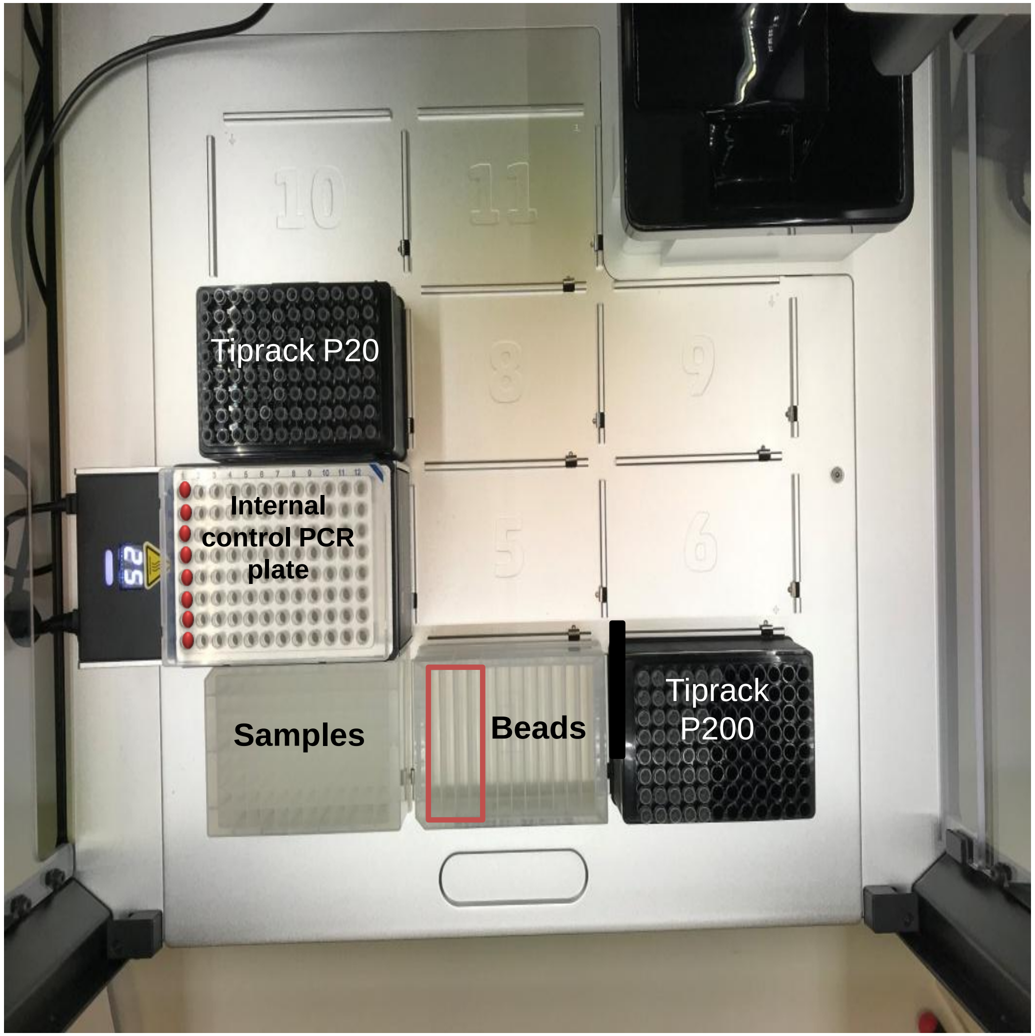

Pipettes
Left: m20
Right: m300

Slots

1. KingFisher DeepWell plate
2. 12 channel NEST reservoir (up to 4 channels for beads)
3. Tiprack 200
4. PCR plate + mod Temp.
5. *Empty*
6. *Empty*
7. Tiprack 20
8. *Empty*
9. *Empty*
10. *Empty*
11. Waste pool reservoir
12. Wastebin

OT2-KF VPII
Station B1

Reagent setup
(plate filling)

KF-B1

Disposition VPII

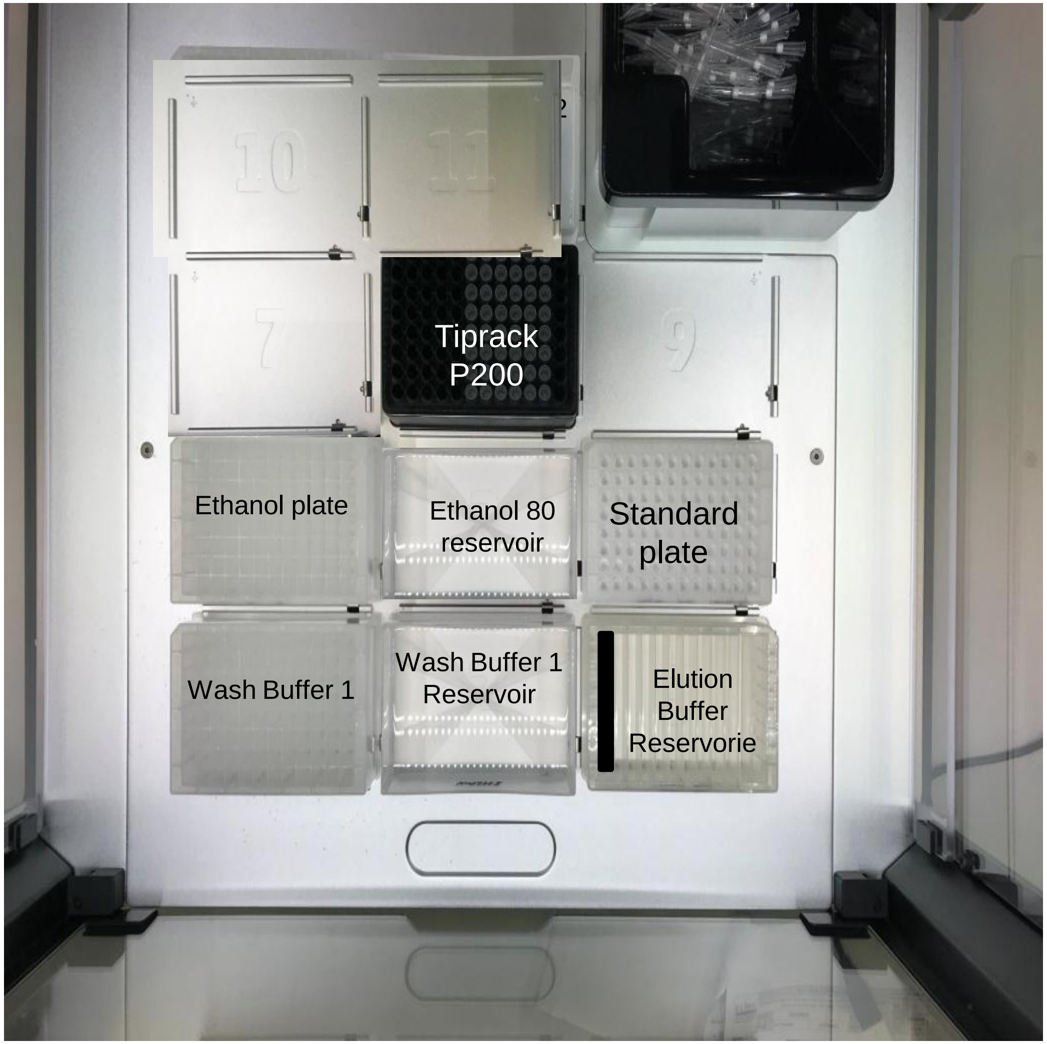

Pipettes
Left: m20 (not used)
Right: m300

Slots

1. KingFisher DeepWell plate
2. 300ml NEST reservoir
3. 12 channel NEST reservoir
4. KingFisher DeepWell plate
5. 300ml NEST reservoir
6. KingFisher Standard plate
7. *Empty*
8. Tiprack 200
9. *Empty*
10. *Empty*
11. *Empty*
12. Wastebin

OT2-KF VPII
Station B2

Sample
preparation

KF-B2

Disposition VPII

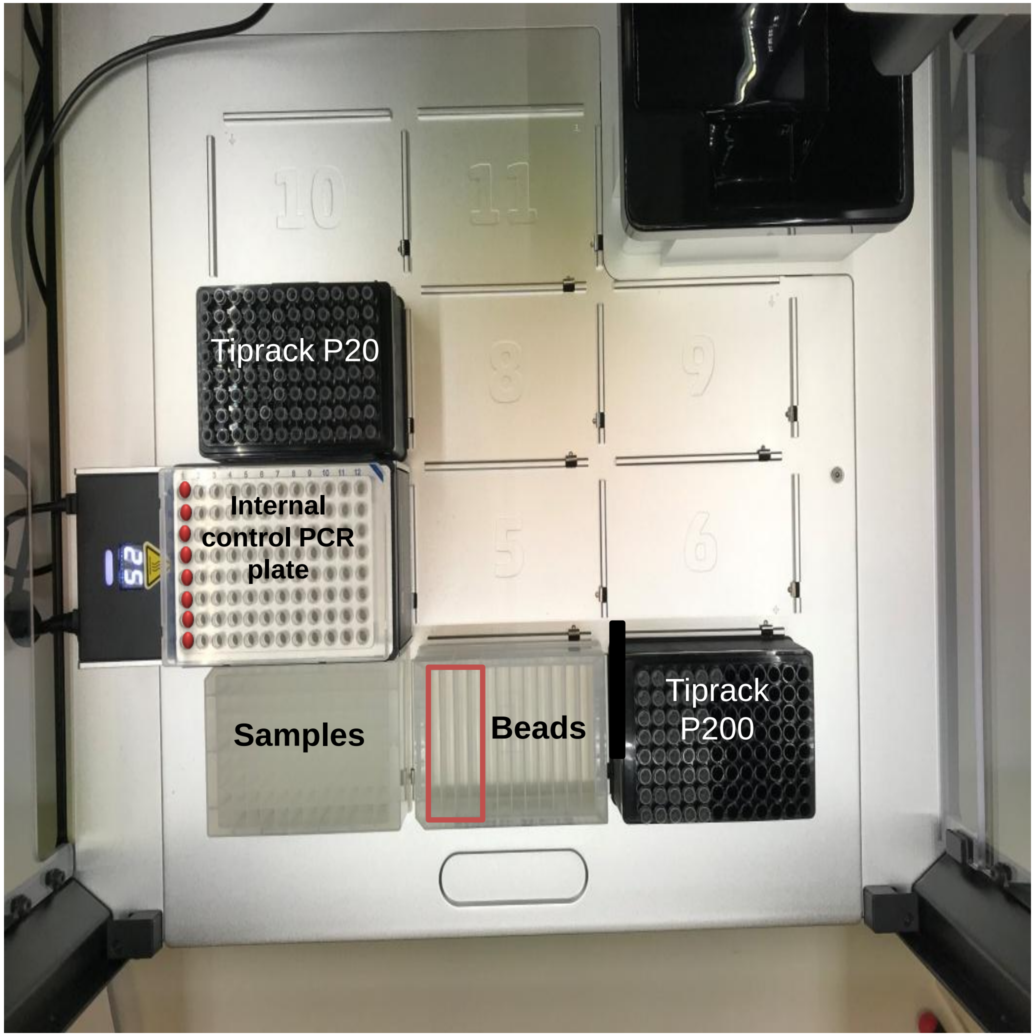

Pipettes
Left: m20
Right: m300

Slots

1. KingFisher DeepWell plate
2. 12 channel Perkinelmer 21mL reservoir
3. Tiprack 200
4. PCR plate + mod Temp.
5. *Empty*
6. *Empty*
7. Tiprack 20
8. *Empty*
9. *Empty*
10. *Empty*
11. *Empty*
12. Wastebin

OT2-KF
Station C

qPCR
setup

KF-C

Disposition

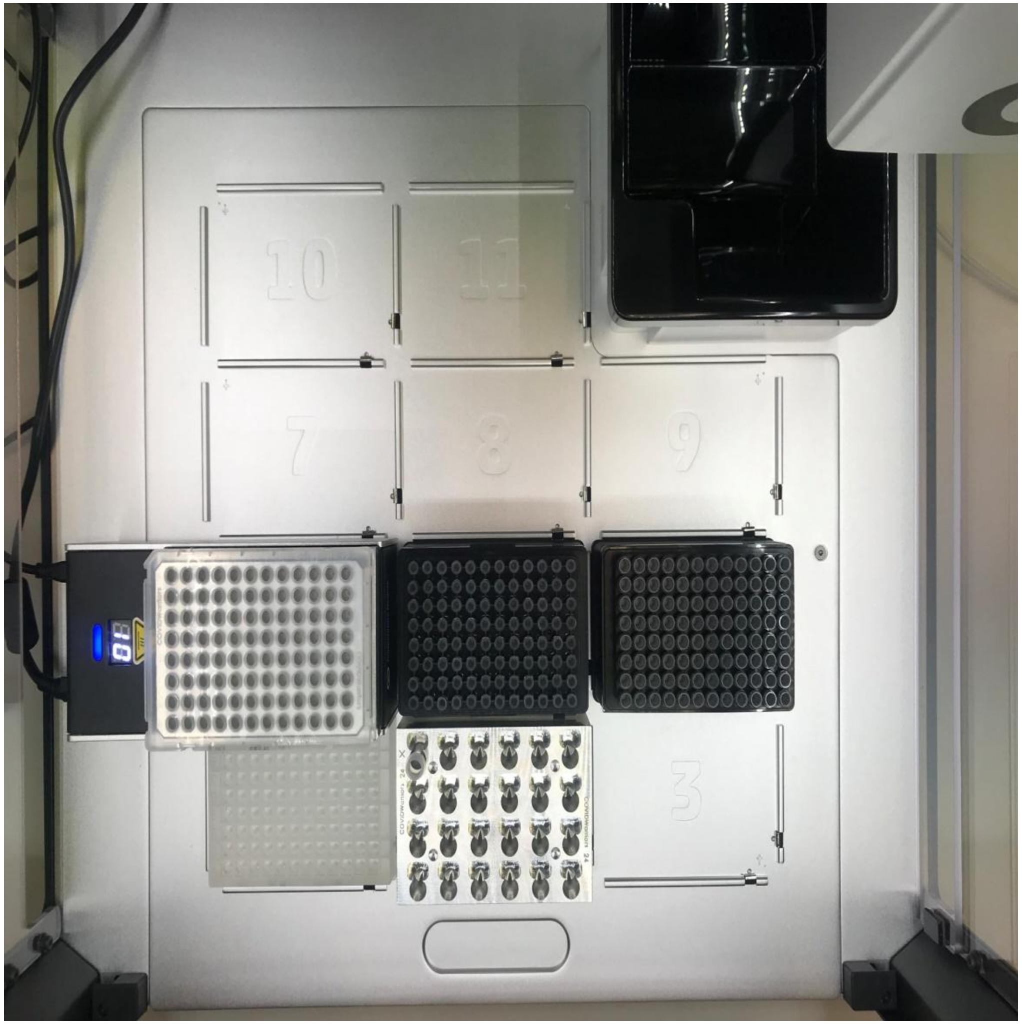

Pipettes

Left: p300
Right: m20

Slots

1. Deepwell
2. Mastermix (2 screwcaps, ref)
3. *Empty*
4. qPCR plate + mod Temp.
5. Tiprack 20
6. Tiprack 200
7. *Empty*
8. *Empty*
9. *Empty*
10. *Empty*
11. *Empty*
12. Wastebin

Pathogen kit

Total material for one run

Reusable
2 Aluminium blocks 96
1 Aluminium block 24
4 racks with eppendorf adaptors
2 Temperature modules

Consumable

1 Tiprack 1000

3 Tiprack 200

2 Tiprack 20

5 KingFisher Deepwell plate

1 Standard KingFisher plate

1 PCR plate 96

1 qPCR plate 96

2 Reservoir 300 mL

2 Reservoir 12 channel NEST

Viral Pathogen II kit

Total material for one run

Reusable

2 Aluminium blocks 96
1 Aluminium block 24
4 racks with eppendorf adaptors
2 Temperature modules

Consumable

1 Tiprack 1000

3 Tiprack 200

2 Tiprack 20

3 KingFisher Deepwell plate

1 Standard KingFisher plate

1 PCR plate 96

1 qPCR plate 96

2 Reservoir 300 mL

1 Reservoir 12 channel NEST

1 Reservoir 12 channel perkinelmer (21ml)
