# Supplementary figures and images for: Implementation of an open-source robotic platform for SARS-CoV-2 testing by real-time RT-PCR
Source: PLoS One. 2021 Jul 14;16(7):e0252509. doi: 10.1371/journal.pone.0252509 (PMC8279358; doi:10.1371/journal.pone.0252509)

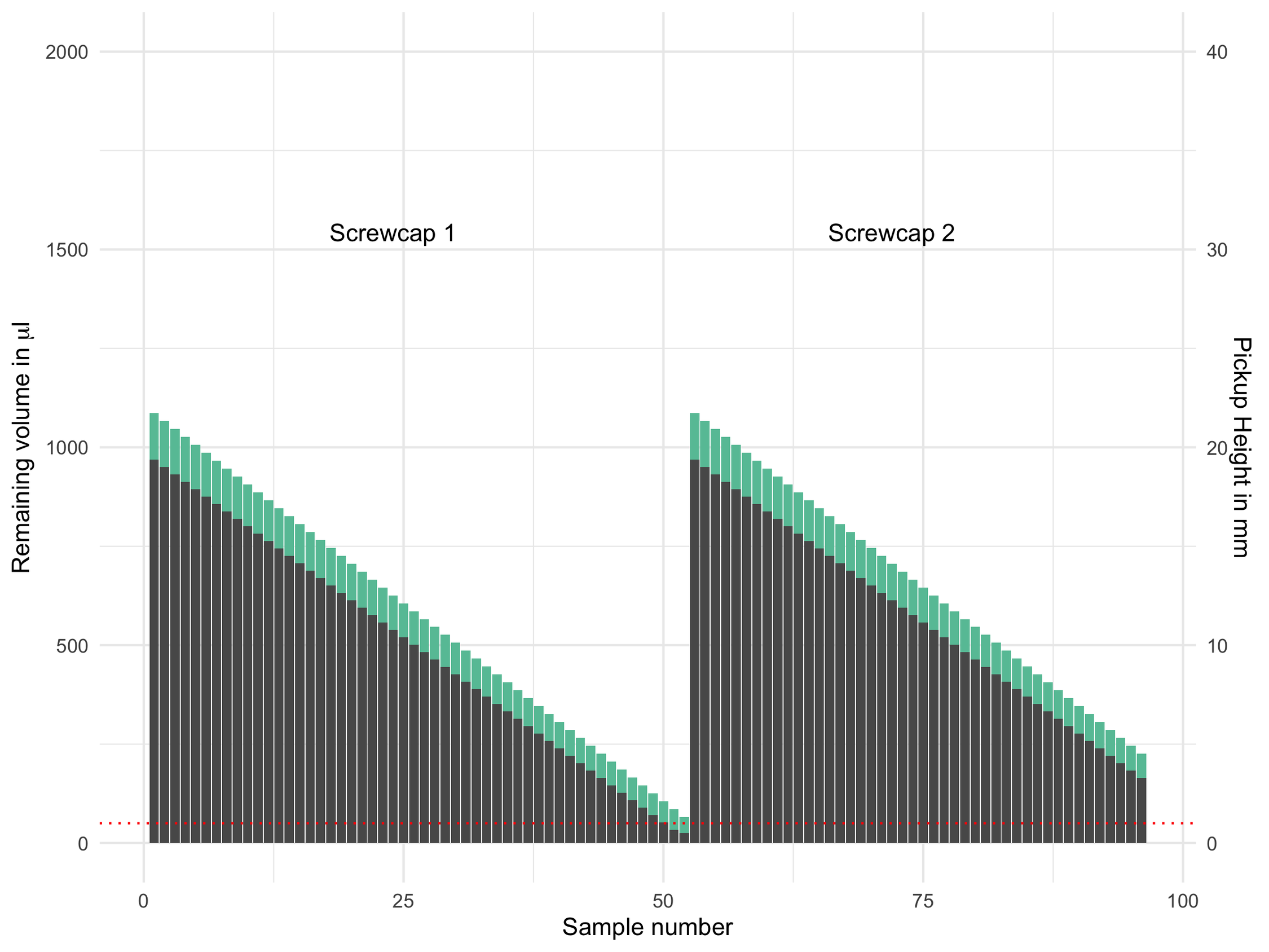

Supplement: S1 Fig — (TIF) [file pone.0252509.s003.tif]
